# Grammar in ‘agrammatical’ aphasia: What’s intact?

**DOI:** 10.1371/journal.pone.0278676

**Published:** 2022-12-06

**Authors:** Han Zhang, Wolfram Hinzen

**Affiliations:** 1 Department of Translation and Language Sciences, Universitat Pompeu Fabra, Barcelona, Spain; 2 Catalan Institute for Advanced Studies and Research (ICREA), Barcelona, Spain; Universite de Geneve, SWITZERLAND

## Abstract

**Background:**

Aphasia following cerebro-vascular accidents has been a primary source of insight for models of language in the brain. However, deviant language patterns in aphasia may reflect processing limitations and cognitive impairment more than language impairment per se.

**Aims:**

We sought to obtain new evidence from spontaneous speech in Broca’s aphasia (BA) for the intactness of grammatical knowledge, operationalized as the preservation of the basic hierarchical structure of syntactic projections.

**Methods & procedures:**

Speech obtained with the AphasiaBank protocol from 20 people with BA, which were independently rated as also being agrammatic, was analyzed and compared to 20 matched non-brain-damaged controls. We quantified (i) marking of Aspect, Tense, and Modality (A-T-M), which are located at specific (high) layers of the syntactic hierarchy and ordered in relation to one another ([M…[T…[A…]]]); (ii) hierarchies of clausal units ([C…[C]]); (iii) discourse markers embedding clauses, located at the highest layer of the hierarchy; and (iv) attachment of adjuncts at different heights of a given hierarchical syntactic structure. Supplementary evidence was obtained from a typology of errors and from pauses subcategorized according to their hierarchical syntactic position.

**Outcomes & results:**

Groups did not quantitatively differ on rates of either Aspect or Modality but underproduced T and embedded clauses. Evidence for compensatory effects was seen in both of the latter two cases. While all adjunct types were underproduced in BA, and pauses overproduced, both showed the same relative proportions within both groups. Errors were largely restricted to omissions, of a kind that would also be expected in condensed neurotypical speech.

**Conclusions:**

Overall, these patterns support the hypothesis of intactness of grammatical knowledge in BA clinically rated as agrammatic, questioning it as a disease model of language impairment.

## Introduction

When searching for the neurobiological basis of language, nothing appears more natural than gleaning insights from how language malfunctions under conditions of brain damage. Unsurprisingly, therefore, aphasia following cerebro-vascular accidents has been a primary disease model of language, informing neuroanatomical models of language to this day [1, 2]. Language dysfunction, however, can as such have multiple causes, as language does not function in isolation but involves the concerted action of numerous neurocognitive systems. These might disturb the normal use of language, even when its basic linguistic mechanisms or representations remain intact. According to the ‘access hypothesis’, specifically, in aphasia ‘language mechanisms are fundamentally preserved and aphasic language behaviours are instead due to impairments of cognitive processes supporting their construction’ ([3], p. 169). This hypothesis is supported through multiple sources of evidence, from the preservation of metalinguistic knowledge in aphasia [4–6], to priming effects [7–9], effects of processing prostheses [10–12], patterns of variability and transiency [3], correlations with specific nonverbal cognitive systems [13], and pervasive nonverbal cognitive impairment in aphasia, as has been consistently documented in group studies [14–17].

Also consistent with the hypothesis of intactness of grammatical knowledge per se is that residual language function in the aphasic brain still seems to largely rely on parts of the pre-existing language network, rather than regions that were not involved in language premorbidly [18]. Griffis et al. [19] showed that a canonical bilateral semantic language network seen in healthy controls still supports semantic processing in people with aphasia (PWA). Ramage et al. [20] demonstrated that functional connectivity in the resting state of brain regions associated with semantic-phonological processing in healthy controls is also predictive of language performance in PWA. A recent study of effective connectivity similarly concludes that ‘language networks in patients with aphasia resemble normal language control networks and that this similarity is accentuated by rehabilitation’ ([21], p. 1). Studies of the dynamics of language reorganization after stroke support this perspective, too, as they indicate a return to function with recovery in core language regions [22].

If and to the extent that language itself is not impaired in aphasia, aphasia will be limited as a disease model of language, though it could illuminate mechanisms involved in access to it. To the same extent, aspects of aphasic speech said to be ‘agrammatic’ may be limited as a way of illuminating the neural basis of grammar and yield a distorted picture of the grammatical competence involved. To understand the neural basis of a true loss of such competence, other disease models would need to be considered, such as those 30% of children and adults with autism spectrum disorder (ASD) who never develop functional language to begin with, and who remain restricted in their linguistic repertoire to no or a handful of single words, with a uniform absence of grammar in production and comprehension [23, 24]. On the other hand, the hypothesis of intactness is by no means uncontroversial and an equally prominent foundational view defines aphasia as language impairment dissociated from cognitive impairment [25].

So far, evidence for or against a version of this broad hypothesis has rarely come from linguistic analyses of spontaneous speech itself, i.e. patterns of specific syntactic constructions spontaneously used or not used by PWA. Spontaneous connected speech and elicitation methods in controlled experiments both have their advantages and drawbacks (see e.g. [26], for issues of potential strategic adaptation in elicitation tasks). While studies based on elicitation tasks (reviewed below) outnumber connected speech studies by far, the latter are more natural and can provide valuable complementary information. However, as reviewed in Prins & Bastiaanse [27] and Bryant et al. [28], so far typical variables in quantitative analyses of spontaneous speech in aphasia have been of a rather generic type, such as sample length (number of utterances), fluency (e.g. speech rate), complexity (e.g. mean length of utterance, percentage of complex utterances, proportion of closed class words, proportion of verbal inflections, embedding index, etc.), and lexical measures (e.g. type-token ratio, word-finding difficulties). Saffran et al. [29] and Rochon et al. [30], for example, report a reduction in verb inflections or the proportion of well-formed sentences in PWA. A recent study on Thai agrammatic spontaneous speech [31], using measures such as utterance length and number and diversity of verbs, has again reached similar findings. Little can be concluded from such variables for the intactness of grammatical knowledge as conceptualized here. Against this background, our principle aim here was to develop a novel annotation scheme for spontaneous speech in aphasia, specifically designed to test the hypothesis of the intactness of linguistic representations.

Any such annotation scheme requires addressing the challenge of how to operationalize the notion of the ‘intactness of linguistic knowledge’ or that of ‘linguistic representations’. A reduction of linguistic complexity and a pattern of formal grammatical errors virtually defines our notion of BA and agrammatism, hence any speech sample of people with BA prima facie falsifies such intactness. The intactness in question thus needs to be operationalized in a way that clear predictions emerge, in particular, for when or under what criteria such evidence of linguistic impairment can be considered as only apparent. Here we based such predictions on the operationalization of the notion of intact grammatical knowledge as the preservation of the *syntactic hierarchy*. Sentences are not merely organized linearly with one word following another, but at the level of phrases that embed other phrases, creating a hierarchy involving asymmetric entailments of some phrases by others. These phrases have abstract types, such that noun phrases (NPs) for example are embedded in verb phrases (VP), and all functional morphemes head phrases stacked hierarchically in a pre-determined order. This is illustrated in *She may be making coffee*, where *may* encodes modality, *be* encodes (non-finite) Tense, and *-ing* encodes Aspect, such that Aspect is embedded in Tense, and Tense in Modality hierarchically: [S [NP *she*] [MOD *may* [T *be* [ASP+VP *mak-ing* [NP *coffee*]]]]] (see e.g. [32], chapter 9, for a textbook account). Reversals of this hierarchy are not known, such as modalities being expressed as part of VP-morphology and in the scope of a higher aspectual morpheme. In a similar way, in English there is a hierarchical relation between Tense and Aspect, in that a VP specified for Aspect with missing Tense cannot make up a sentence and Tense necessarily has Aspect in its scope when both occur. In general, as we ‘climb up’ the syntactic tree [33], higher levels entail more complexity. The specific layers of that complexity have already been proposed to illuminate the acquisition sequence [34] and aphasia alike [33].

A great number of studies have already targeted Tense in aphasia and suggested that tense morphology is specifically impaired in BA across different production tasks, such as sentence completion and repetition (in Spanish and English: [35]; in Hebrew and in Arabic: [36]; in German: [37]; in English: [38]; in Dutch: [39]). Evidence from spontaneous speech is very sparse, however (for some early case studies, see [40–42]), and to our knowledge, Aspect, Tense and Modality have not yet been looked at in conjunction and in relation to one other. This could also address the question whether their relative proportions form a neurotypical pattern or not. Therefore, the A-T-M complex formed our first set of variables here.

Next, we explored the formation of hierarchies at the level of full clauses (complementizer phrases or CPs), as in [CP *He* [VP *thinks* [CP *it is cold outside*]). Quantitative analysis of spontaneous speech in BA has previously revealed reduced production of embedded clauses [29, 36]. However, there is also evidence that PWA tend to use more reported speech in their narratives [43–45], indicating a potential compensation for difficulties in clausal embedding through quotational embedding. We therefore quantified both forms of embedding and investigated their relation to each other.

The third dimension of interest consisted in grammatical markers of discourse and interactional language, such as confirmationals and response markers, based on evidence that these, too, are subject to grammatical constraints at the utterance level, governing a universal layer of grammatical structure above CP [46, 47]. For example, tag-question in English such as *isn’t it* regulate hearer agreement to what is being said. They cannot attach to structural configurations that could not be utterances, e.g. *It to be there, *isn’t it*, as opposed to *It is there*, *isn’t it*, or *Shining, *isn’t it*, as opposed to *The sun is shining*, *isn’t it*, or *Sunny*, *isn’t it*. All human languages harbor a range of specific grammatical devices regulating aspects of linguistic interaction such as what speech act type is being made [48, 49], which epistemic attitude is being expressed [50, 51], how a proposition is embedded in the discourse and speech context [52, 53], etc. Wiltschko [47] conceptualizes such devices as the ‘grammar of interaction’ and suggests a specific zone at the top of the syntactic hierarchy to be dedicated to regulate such interactive aspects of language.

As a fourth way of addressing the intactness of the syntactic hierarchy, we considered adjunct placement, as adjuncts of different types inherently attach to the syntactic tree at different heights. Thus, low-scope adverb such as *quickly* or *twice* are attached to (or modify) verb phrases (VP), qualifying aspects such as the manner or frequency in which a given action is performed. A higher-scope adverb like *surprisingly*, on the other hand, inherently scopes over an entire already specified proposition (e.g., *he won the lottery twice*), qualifying the subjective probability with which a given event occurred, and hence implying a more projected syntactic tree. In between them there are adjuncts taking intermediate scope, such as *last year*, hence yielding three hierarchically different attachment sites in an utterance like *Surprisingly*, *he won the lottery twice last year*. To our knowledge, no study has so far systematically examined the hierarchy of adjunct placement in aphasia (though there is some evidence that temporal adjunct placement is relatively preserved in the speech of PWA: [54]).

The fifth dimension of interest was pauses, which can address the syntactic hierarchy insofar as they are considered together with the syntactic positions in which they appear. Thus, a pause ahead of a grammatical junction position, such as ahead of an utterance or between the subject and the predicate of a sentence, can be interpreted as indicative of planning for grammatically assembling an utterance, while a pause ahead of a lexical noun after the determiner *the*, or between an auxiliary and a lexical verb, plausibly indicates a lexical retrieval problem. While pauses in positions ahead of grammatically complex units may indicate awareness of the grammatical constraints involved, pauses appearing randomly with regards to grammar would indicate the opposite. While we generally expect an increase in pausing in aphasia due to the processing difficulties involved, a potential similarity between PWA and controls in the distribution of pauses across such different positions in the syntactic hierarchy, and in relation to one another, could thus further address neurotypical aspects of aphasic language. While there are relatively few studies in this regard, Angelopoulou et al. [55] recently already suggested that PWA exhibited a qualitatively similar pattern of pause distribution compared to neurotypical speakers. However, their evidence is not easy to interpret as a mixed group with different types of aphasia was used, filled pauses were not included, and syntactic pause positions were not subclassified as occurring within-phrases (ahead of a lexical retrieval position), between-phrases, or between-utterances.

Sixth and finally, we included an analysis of error patterns. It has already been documented that errors often arise from omissions in PWA [29, 30]. Such errors, even when they lead to formally incomplete or ungrammatical sentences, can be consistent with an error-avoidance strategy and hence the grammatical competence implicit in the awareness of what would be an error. Moreover, omissions in a language such as English could be those where material is omitted that even neurotypical speakers might omit in resource-limiting circumstances [56], hence indicating no loss of grammatical competence. Thus, when writing a telegram, neurotypical speakers might drop determiners, auxiliaries and other grammatical material, when the lexical material provided and context make these omitted items sufficiently predictable and the intended propositions inferable. In many languages, such devices do not exist to begin with, suggesting they reflect language-specific rather than universal grammatical constraints. The same applies to morphological errors, which would not even exist in languages without inflectional morphology. A less expected type of error in neurotypical telegraphic speech, on the other hand, would be a word order error, since it is unclear which resource limitation would lead for a preference of one word order over another. We thus subclassified errors into omissions (including both free and bound morphemes), morphological errors, and word order violations.

Our overarching hypothesis was that grammatical competence is fundamentally intact in BA. We addressed this hypothesis by testing the following specific predictions in the context of the above annotation scheme:

1. Speakers with BA will significantly produce *each* functional morphemes of the ATM complex and do so in similar *proportions* as their neurotypical matches, when controlling for speech quantity. Significant differences in the *relative* proportions of A, T, and M, moreover, when these are compared to each other *within* each group, will be similar in both groups.
2. PWA will produce similar levels of clausal embedding as controls, with a possible compensation effect seen at the level of quotational embedding.
3. PWA will produce the grammar of interactive language similarly as controls.
4. PWA will produce adjuncts at all heights of the syntactic hierarchy distinguished here, and significant differences in the relative proportions of the three adjunct types will match those seen in controls.
5. Pausing patterns in PWA will predominantly reflect planning for grammar and be similar to the patterns in neurotypical controls.
6. Error patterns in argument relations will predominantly involve omissions.

Importantly, we addressed these predictions in those PWA where they *can* be addressed, i.e. whose productions reach a relevant threshold of grammatical complexity. In particular, productions with zero instances of any word combinations would immediately falsify all of our predictions. We therefore restricted our study to those people with BA whose productions do break into grammar to an extent that the above predictions can be addressed. While our results therefore apply to a clinically prominent instance of BA, they do not cover the full range of BA, let alone of aphasia.

## Methodology

### Participants

Participants data were obtained from the AphasiaBank database (https://aphasia.talkbank.org/) [57], which is a shared database of multimedia interactions for the study of communication in aphasia publicly available for researchers worldwide. When the data were collected, participants signed a written informed consent form which was approved by the local IRB of data contributors. The use and analysis of data for the present study was in adherence to the TalkBank Code of Ethics (https://talkbank.org/share/ethics.html). A total of 40 individuals were included in this study, including 20 persons with Broca’s aphasia (BA: mean age: 56;5, range: 31;8–81;4 years old) and 20 healthy controls (HC: mean age: 58;2, range: 33;6–81;0 years old). The inclusion criteria for people with aphasia were (1) a clinical classification of the aphasia type as BA, determined based on Western Aphasia Battery (WAB; [58]); (2) an aphasia quotient (AQ) above 51 in the WAB tests. The AQ is a summary score that indicates the overall severity of language impairment, where 51–75 is moderate and >76 is mild; (3) a capacity for producing at least three-words combinations; (4) a single stroke resulting in left hemisphere lesions; (5) at least six months post-stroke onset; (6) English as primary language; (7) premorbidly right-handed.

Since a diagnosis of BA is not sufficient to signify that a patient has agrammatism [59], the videos of speech production of our subjects from AphasiaBank were re-rated by an expert clinical neurologist who is a specialist in primary progressive aphasia and was blind to the aims of the study. He was asked to independently rated each subject as being agrammatic or not, following the procedure of classifying agrammatism and paragrammatism specified in Matchin et al. [60], based on the videos of speech production of the subjects from AphasiaBank. Of the 20 BA patients in our study, all were confirmed as agrammatic. This classification was further consistent with results as documented here, in particular error types (which largely consisted of omissions), as well as mean utterance length, which was significantly shorter in the BA group than in controls (BA = 10.76, HC = 29.20, p < .001). Stroke patients were matched for age, years of education with 20 healthy controls that had English as their primary language and no history of stroke, head injury or neurological condition. See Table 1 for details of the participants’ demographic information.

**Table 1:** Participant information.

	BA (n = 20)	HC (n = 20)	t	p
Age of testing	56;5 (31;8–81;4)	58;2 (33;6–81;0)	-0.474	0.638
Years of education	15 (12–20)	16 (12–20)	-1.412	0.166

### Speech samples and transcription

Speech samples consisted of participants’ responses to structured interviews and picture description tasks obtained from AphasiaBank [57]. Structured interviews involved the participants recalling the stroke experience (or an injury story in the case of healthy controls), coping with the condition, and an important event that molded their life. The picture description included three stimuli: cat rescue [61]; broken window and refused umbrella [57]. All speech samples within the AphasiaBank database are transcribed in the CHAT format [62].

### Annotation

Each participant’s speech transcription and its corresponding media file were imported to ELAN (https://archive.mpi.nl/tla/elan), where the annotations were carried out. The basic units of analysis were ‘meaningful grammatical units’ (‘g-units’). We allowed these to involve grammatical errors, yet they needed to exhibit a clear indication of a grammatical connection and a meaning assigned to the construction as a whole, determinable either from the construction itself or the construction plus context including gestures (e.g. *I have sell my boat; Now let’s me finish; My father has blind*). Such units were then further subdivided into sentential and non-sentential utterances. We excluded formulaic utterances (e.g. *I don’t know*, *I’m sorry*), when multiply repeated, in our analysis, considering that the inclusion of them could lead to a distortional inflation of grammatical complexity and number of g-units.

We subsequently annotated which elements of grammaticality these units exhibited along the layers of the syntactic hierarchy. For a full list of variables, formal definitions and examples, see S1 Table in S1 File. In summary, within sentential g-units, we coded: (1) Instances of marking of Aspect-Tense-Modality (counted separately). For all g-units, we coded (2) Quotational and Non-quotational embedding, (3) Markers of interactional language (including Response markers and Confirmatioanls), (4) Adjuncts of different types, and (5) Pauses of different types depending on the syntactic position in which they occurred.

First, with respect to Aspect-Tense-Modality, we annotated all explicit markers of Aspect (e.g. *was driving*, *used to have*, *about to go*, etc.), temporal displacement (e.g. past tense), and Modality (*can*, *may*, *should*, *will*, etc.). As a further measure of clausal complexity, we annotated clausal embedding, distinguishing between Quotational and Non-quotational embedding. With respect to the former, in addition to the utterances that were already transcribed with quotation markers, we also annotated quotation when participants clearly indicated through intonation or context that they were quoting the utterance of some person. Aspects of interactional language annotated were Response markers and Confirmationals. We only annotated markers of interactional language attached to g-units, ignoring those that stood on their own.

Adjuncts were classified according to which layer of grammatical hierarchical complexity above the NP they targeted, specifically identifying whether they were adverbials attached to VPs modifying the event/action denoted by the verb (e.g. *slowly*, *eagerly*), or else adjoined to larger units such as clauses. These ‘non-VP related’ adjuncts were further split as having scope either within a propositional (truth-evaluable) unit (‘Within-propositional adjunct’), or outside of such a unit, hence a larger scope (‘Outside-propositional adjunct’). Examples of the former were temporal adjuncts like *in the afternoon*, *five years ago*, etc. Examples of the latter were adjuncts expressing the speaker’s evaluation of a proposition (e.g. *Sadly*, *Surprisingly*, *Stupidly*), or his/her epistemic or evaluative relations to a proposition (e.g. *Actually*, *Probably*, *Really interesting*!, *Amazing*!).

We divided pauses into three types according to the syntactic position in which they occurred, identifying whether they occurred in a boundary position of the g-unit (specifically, at the beginning) or within the g-unit. In the latter case, we further distinguished between ‘Between-phrase pauses’ (e.g. between the subject and the predicate) and ‘Within-phrase pauses’ (e.g. between the determiner and the noun in NPs; between the auxiliary and the verb; between the preposition and the NP/VP; between the copula and the adjective/noun; between the main verb and the object; or between the adverb and the verb).

Lastly, for the analysis of formal grammatical errors within sentential g-units, we distinguished among three different error types, including (1) Omissions (both free and bound morphemes), (2) Morphological errors, and (3) Word order violations (for a full list of error types, definitions, and examples, see S2 Table in S1 File).

### Reliability

To establish the inter-rater reliability of coding, 25% of the data were randomly selected and recoded by an independent rater who was not involved in this study. Independent rating samples were checked against the original ratings on a point-to-point basis and disagreements were attempted to be resolved by discussion to reach consensus from the two raters. Final reliability was calculated for all variables by dividing the total number of points the two ratings agreed by the sum of the total points possible, resulting in 97% reliability.

### Statistical analysis

Statistical analysis was carried out in R (https://www.R-project.org/) and was divided into three stages dealing with group comparisons, within-group analysis and error analysis, respectively. First, a set of mixed effects negative binomial regression models using the glmmTMB package, version 1.0.2.1 [63] in R were fitted to evaluate potential group differences in the incidence rate of 13 linguistic variables from five domains, namely ATM, Embedding, Interactional language, Adjuncts, Pauses, which were defined as predicted variables in the models. Mixed effects negative binomial regression is a generalization of the Poisson mixed-effects model which works on repeated count data. It is usually used for modeling over-dispersed count outcome variables, as it loosens the restrictive assumption that the variance is equal to the mean made by the Poisson mixed-effects model. Each model included a fixed effect of Group as a categorical predictor, a random intercept for subject, and an offset term containing the total number of g-units (or sentential g-units in the cases of ATM) to account for the variation in the number of g-units each participant produced. This served to adjust for the amount of opportunity an event of interest has for occurring, and it changed the predicted variable from a count into a rate.

Stage two concerned the within-group analysis of the two groups. Due to the non-normal distribution of the data, non-parametric Friedman tests were carried out to examine the potential differences in the mean ratios of the subdivision variables within the domain of ATM, Adjuncts and Pauses. A significant Friedman test was then followed up by pairwise Wilcoxon signed-rank tests, and p-values were adjusted using the Bonferroni multiple testing correction method, by multiplying p-values by the number of comparisons. All indicated p-values were reported in their already corrected form and the significance level was set at 0.05. The Kendall’s W was applied as the measure of the Friedman test effect size. Kendall’s W uses the Cohen’s interpretation guidelines of 0.1 - < 0.3 (small effect), 0.3 - < 0.5 (moderate effect) and > = 0.5 (large effect). Further, Paired Wilcoxon signed-rank tests were performed to determine whether there was a difference in the mean ratios of (1) Quotational vs. Non-quotational embedding and (2) Response markers vs. Confirmationals. To take speech quantity into account, the aforementioned variables were converted into ratios by dividing them either by the total number of g-units or by the total number of sentential g-units (in the case of ATM).

Lastly, concerning the aphasic error analysis, we used descriptive statistics to display the mean and standard deviation of the three types of errors out of the total number of errors a participant produced.

## Results

### Description of the speech sample

The mean and standard deviation for the total number of g-units produced by the clinical group were 53.30 and 14.84, respectively; for Sentential g-units 32.60 and 20.02, respectively; and for Non-sentential g-units 18.40 and 11.18, respectively. The mean and standard deviation for the total number of g-units produced by the control group were 51.00 and 17.14, respectively; for Sentential g-units 50.20 and 14.38, respectively; and for Non-sentential g-units 3.10 and 3.18, respectively.

### Between-group comparisons

**Fig 1:**
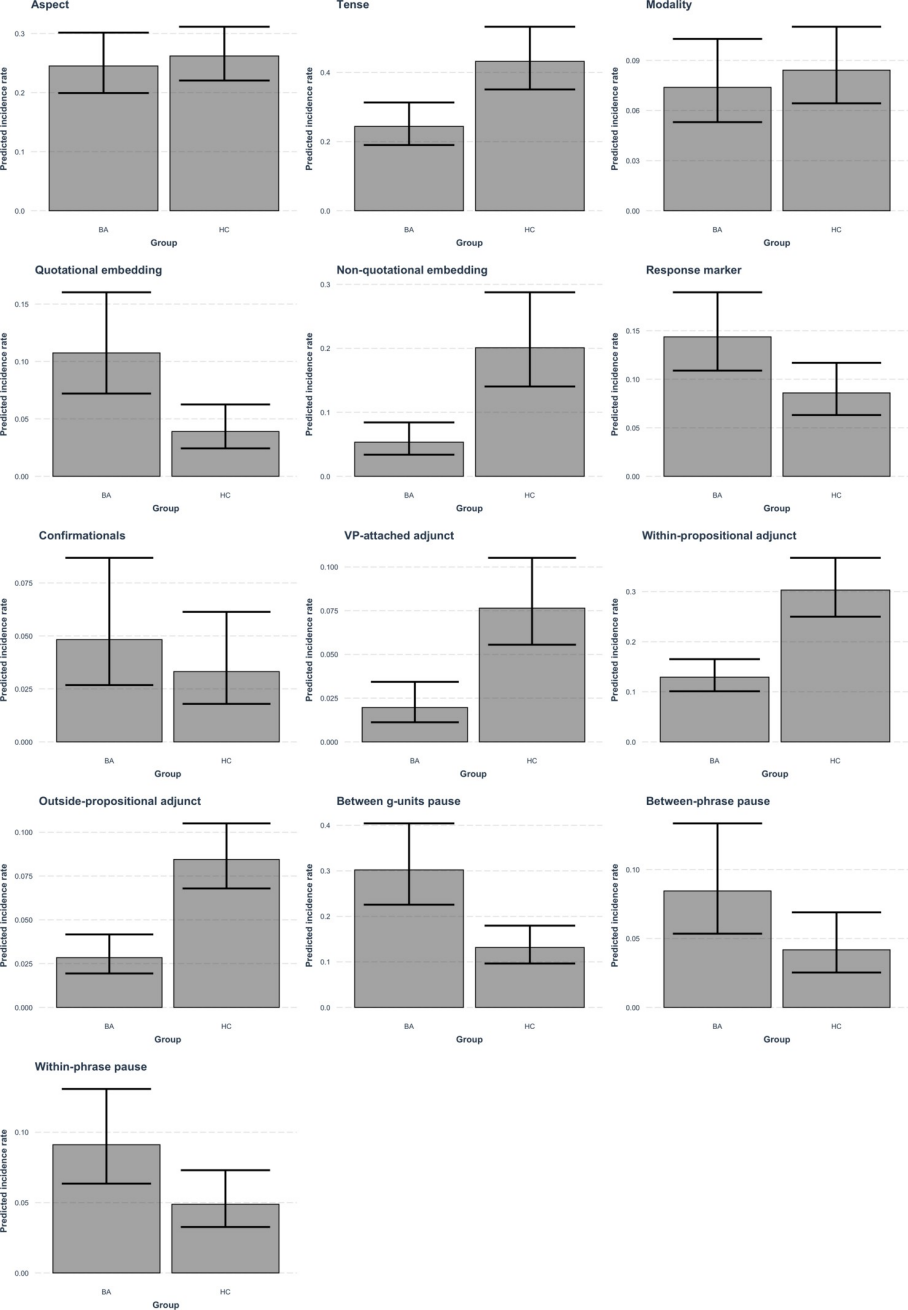
Estimated rates of the 13 linguistic variables for the two groups.

A summary of results from the mixed effects negative binomial regression models is presented in Table 2, where the exponentiated model coefficients represent the incidence rate ratios of the 13 linguistic variables produced by the groups, in which BA was defined as the reference group. The results show that no significant differences were found in the estimated rates of Aspect, Modality, Confirmationals, Between-phrase pauses, and Within-phrase pauses between groups. However, compared to BA, healthy controls had significantly higher rates of Tense, of Non-quotational embedding, and of Adjunct of all types, while the probability of Quotational embedding, of Response marker, and of Between g-units pauses occurring per g-unit was significantly higher in the clinical group. Fig 1 plots the estimated rates of the 13 linguistic variables for the two groups.

**Table 2:** Statistical significance for possible group differences in the 13 linguistic variables.

	*Fixed effect*							*Random effect*		
*Linguistic Variable*	*Model*	*Predictors*	*Incidence Rate Ratios*	*CI*	*Statistic*	*p-values (Intercept)*	*p-values*	σ^2^	τ_00_	ICC
Aspect	GLMM:NB	BA (Intercept)	0.24 ***	0.20–0.29	-14.27	<0.001		0.09	0.06	0.39
		HC	1.07	0.83–1.38	0.52		0.601			
Tense	GLMM:NB	BA (Intercept)	0.25 ***	0.17–0.36	-7.25	<0.001		0.14	0.08	0.36
		HC	1.74 **	1.20–2.52	2.94		**0.003**			
Modality	GLMM:NB	BA (Intercept)	0.07 ***	0.05–0.10	-14.37	<0.001		0.34	0.15	0.30
		HC	1.13	0.73–1.73	0.54		0.587			
Quotational embedding	GLMM:NB	BA (Intercept)	0.09 ***	0.06–0.15	-9.62	<0.001		0.62	0.28	0.32
		HC	0.37 ***	0.20–0.68	-3.21		**0.001**			
Non-quotational embedding	GLMM:NB	BA (Intercept)	0.05 ***	0.03–0.08	-15.22	<0.001		0.48	0.00	
		HC	4.30 ***	2.48–7.48	5.18		**<0.001**			
Response markers	GLMM:NB	BA (Intercept)	0.13 ***	0.10–0.17	-15.08	<0.001		0.16	0.21	0.56
		HC	0.57 **	0.39–0.85	-2.76		**0.006**			
Confirmationals	GLMM:NB	BA (Intercept)	0.04 ***	0.02–0.07	-10.34	<0.001		0.98	0.37	0.27
		HC	0.70	0.31–1.57	-0.87		0.387			
Vp-attached adjunct	GLMM:NB	BA (Intercept)	0.02 ***	0.01–0.03	-16.57	<0.001		0.44	0.00	
		HC	4.02 ***	2.27–7.11	4.77		**<0.001**			
Within-propositional adjunct	GLMM:NB	BA (Intercept)	0.13 ***	0.10–0.16	-16.54	<0.001		0.17	0.00	
		HC	2.45 ***	1.84–3.26	6.12		**<0.001**			
Outside-propositional adjunct	GLMM:NB	BA (Intercept)	0.03 ***	0.02–0.04	-19.13	<0.001		0.31	0.02	0.07
		HC	3.00 ***	1.95–4.61	4.99		**<0.001**			
Between g-units pauses	GLMM:NB	BA (Intercept)	0.26 ***	0.18–0.36	-7.96	<0.001		0.38	0.22	0.36
		HC	0.49**	0.30–0.81	-2.76		**0.006**			
Between-phrase pauses	GLMM:NB	BA (Intercept)	0.07 ***	0.04–0.11	-10.44	<0.001		0.66	0.34	0.34
		HC	0.58	0.30–1.13	-1.60		0.110			
Within-phrase pauses	GLMM:NB	BA (Intercept)	0.08 ***	0.06–0.13	-12.50	<0.001		0.51	0.21	0.29
		HC	0.57 *	0.33–1.01	-1.94		0.053			

Abbreviations: CI: Confidence Intervals, NBRM: Negative Binomial Regression Model.

* p<0.05

** p<0.01

*** p<0.001

### Within-group analyses

Results from the Friedman and the Wilcoxon tests are summarized in Table 3. In the domain of ATM, Friedman tests suggested that, within both groups, relative mean ratios of Aspect, Tense, and Modality markers differed from each other (BA: p < 0.001, HC: p < 0.001). Specifically, of the ATM complex, both groups produced Modality markers least.

**Table 3:** Statistical significance for clinical and control groups in different subtypes of variables.

*Linguistic variable*		*Mean ± SD*		*Test*	*p-values*		*Effect size*		*PWC*	*p-values*	
		*BA*	*HC*		*BA*	*HC*	*BA*	*HC*		*BA*	*HC*
ATM	Asp	0.260 ± 0.128	0.265 ± 0.087	Friedman	**< 0.001**		0.579		Asp-Tns	1	0.051
	Tns	0.228 ± 0.120	0.425 ± 0.216			**< 0.001**		0.836	Asp-Mod	**<0.001**	**<0.001**
	Mod	0.066 ± 0.053	0.084 ± 0.048						Tns-Mod	**<0.001**	**<0.001**
Embedding	QE	0.106 ± 0.089	0.037 ± 0.042	Wilcoxon	**0.039**	**< 0.001**	0.468	0.835	-	-	-
	NonQE	0.051 ± 0.070	0.202 ± 0.104								
IL	RM	0.147 ± 0.087	0.088 ± 0.060	Wilcoxon	**< 0.001**	**< 0.001**	0.747	0.798	-	-	-
	Conf	0.048 ± 0.060	0.032 ± 0.047								
Adjunct	VpA	0.020 ± 0.030	0.075 ± 0.042	Friedman	**< 0.001**		0.551		VpA-WpA	**<0.001**	**<0.001**
	WpA	0.129 ± 0.090	0.300 ± 0.088			**< 0.001**		0.775	VpA-OpA	0.732	0.615
	OpA	0.027 ± 0.027	0.087 ± 0.037						WpA-OpA	**0.002**	**<0.001**
Pause	BgP	0.303 ± 0.189	0.130 ± 0.092	Friedman	**< 0.001**		0.668		BgP-BpP	**<0.001**	**<0.001**
	BpP	0.085 ± 0.087	0.042 ± 0.046			**< 0.001**		0.543	BgP-WpP	**<0.001**	**0.002**
	WpP	0.090 ± 0.078	0.048 ± 0.040						BpP-WpP	1	1

Abbreviations: PWC: pairwise comparison, Asp: Aspect, Tns: Tense, Mod: Modality, QE: Quotational embedding, NonQE: Non-quotational embedding, IL: Interactional language, RM: Response markers, Conf: Confirmationals, VpA: VP-attached adjunct, WpA: Within-propositional adjunct, OpA: Outside-propositional adjunct, BgP: Between g-units pauses, BpP: Between-phrase pauses, WpP: Within-phrase pauses.

In terms of clausal embedding, the clinical group produced significantly more Quotational than Non-quotational embedding (p = 0.039). By contrast, the control participants generated significantly more Non-quotational than Quotational embedding (p < 0.001). In the domain of interactional language, Paired Wilcoxon signed-rank tests showed that both groups produced significantly more Response markers than Confirmationals (BA: p < 0.001, HC: p < 0.001).

For both groups, the mean ratios for the three Adjunct types were statistically significantly different from each other (BA: p < 0.001, HC: p < 0.001), with the mean ratio of Within-propositional adjuncts being significantly higher than that of the VP-attached (BA: p < 0.001, HC: p < 0.001) and Outside-propositional adjuncts (BA: p = 0.002, HC: p < 0.001).

**Fig 2:**
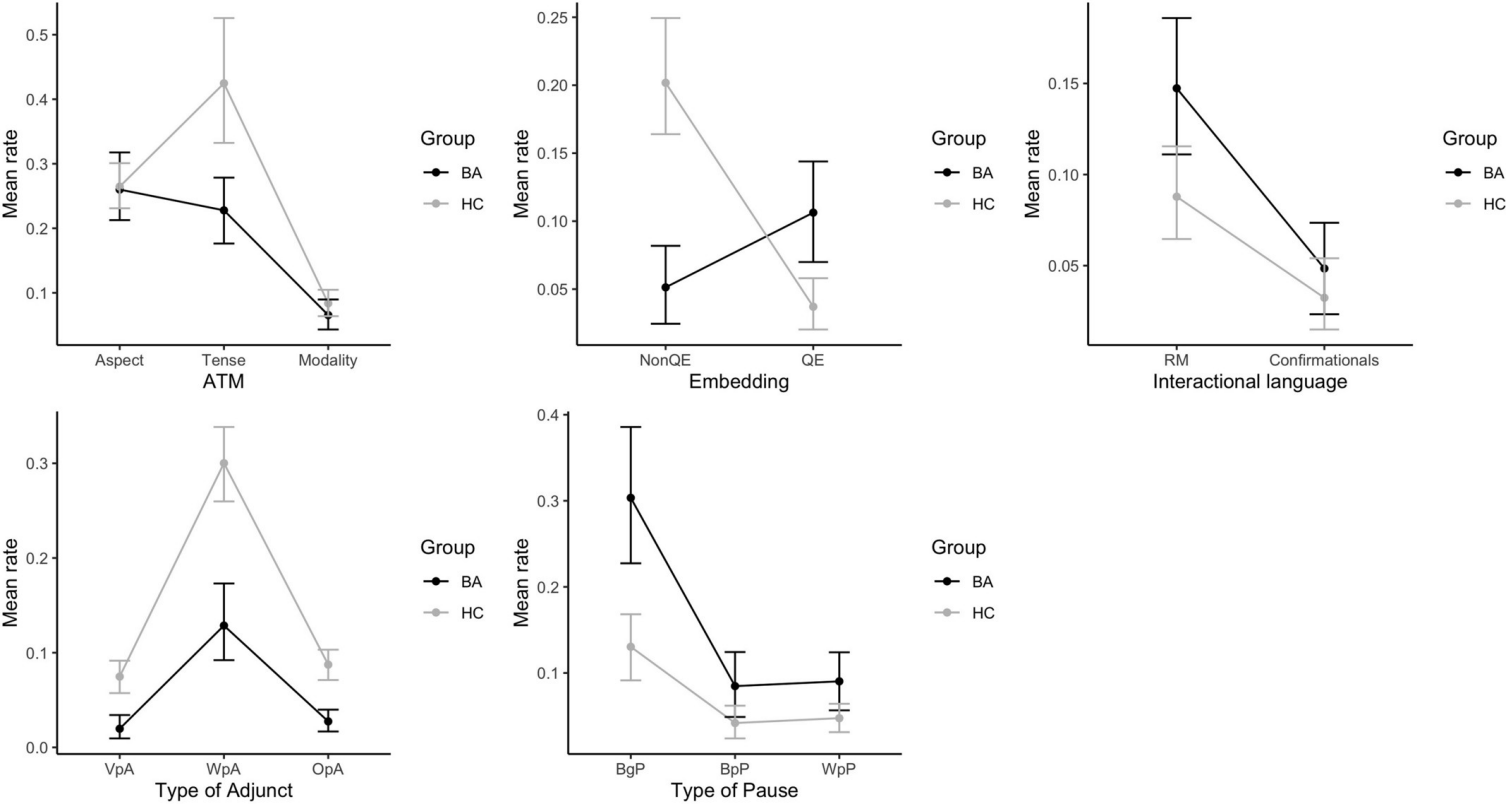
Mean ratios of subtype variables from different domains in both groups. Abbreviations: NonQE: Non-quotational embedding, QE: Quotational embedding, RM: Response markers, VpA: VP-attached adjunct, WpA: Within-propositional adjunct, OpA: Outside-propositional adjunct, BgP: Between g-units pauses, BpP: Between-phrase pauses, WpP: Within-phrase pauses.

Regarding Pauses by syntactic location, Friedman tests revealed a significant difference in the mean ratios between different types of pauses for both groups (BA: p < 0.001, HC: p < 0.001), with the ratio of the Between g-unit pauses being significantly higher than that of both the Between-phrase (BA: p < 0.001, HC: p < 0.001) and the Within-phrase pauses (BA: p < 0.001, HC: p = 0.002). Fig 2 plots the mean ratios of subtype variables from different domains in both groups.

### Error analysis

**Fig 3:**
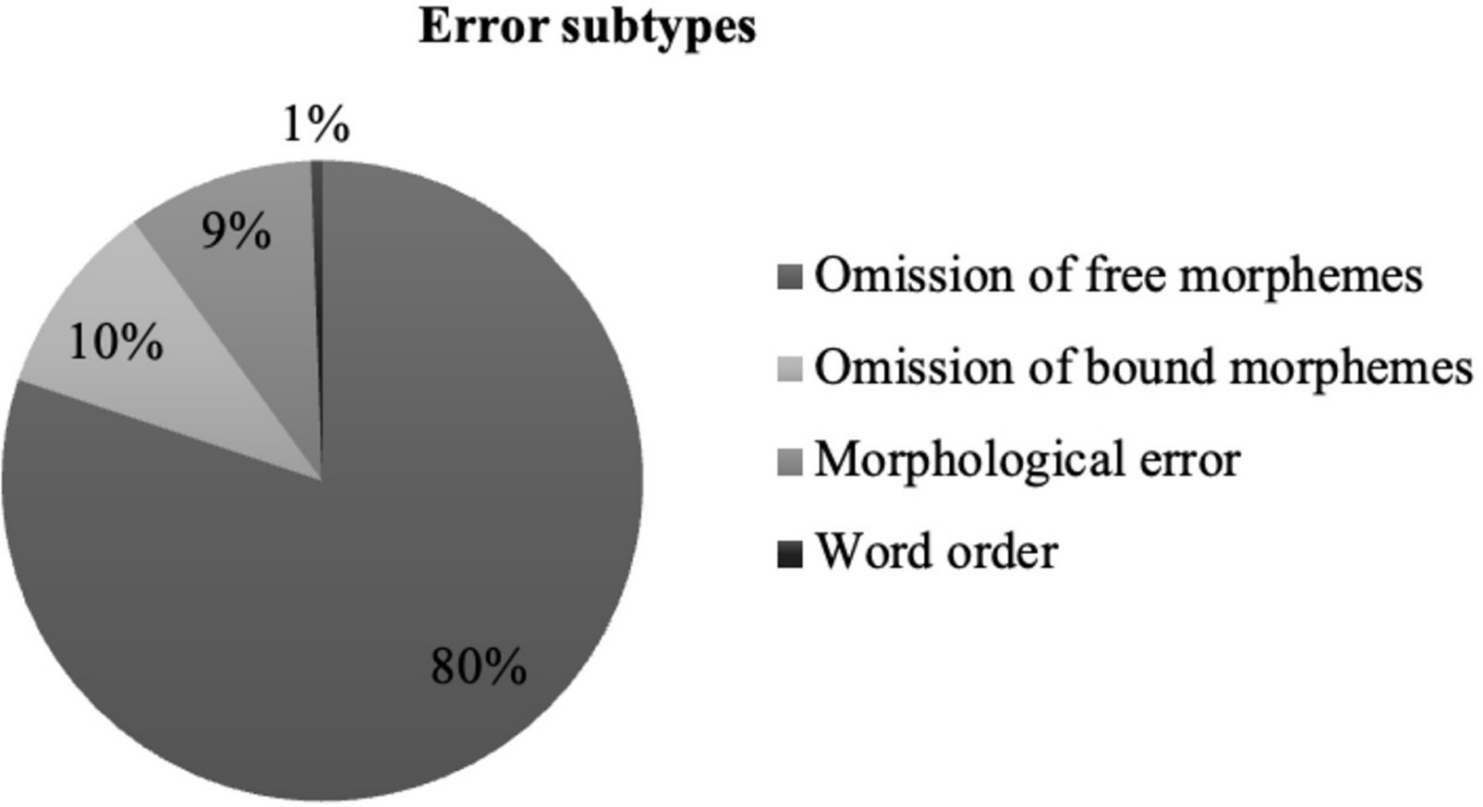
Distribution of error subtypes.

Table 4 shows the mean and standard deviation of the three types of errors we distinguished. Around 90% errors were produced from Omissions (consisting of 80% free morphemes and 10% bound morphemes), 9% from Morphological errors, and only 1% from Word order violations (see Fig 3).

**Table 4:** Descriptive statistics of error subtypes.

Error subtype	Mean ± SD
Omission of free morphemes	0.801 ± 0.239
Omission of bound morphemes	0.099 ± 0.171
Morphological error	0.094 ± 0.113
Word order violation	0.006 ± 0.018

#### Post-hoc analyses

Since the above between-group comparisons of Aspect-Tense-Modality revealed a significant group difference in the use of Tense markers, and given a previous finding that temporal adjuncts tend to be preserved in the speech of speakers with BA [54], we conducted a post-hoc analysis to test the hypothesis that PWA might employ temporal adjuncts as a compensatory strategy to overcome their difficulties in the production of tense morphology. This would suggest, in relation to our overarching hypothesis, that the problem does not relate to Tense or temporal anchoring per se. We thus decomposed the initial Within-propositional adjuncts into Spatiotemporal and Non-spatiotemporal ones, and further extracted all temporal adjuncts indicating time reference to the past from the former group. Afterwards, we computed the ratio of Adjuncts with Past reference (Adjunct Past) to Tense marking for each participant. A Wilcoxon rank-sum test was run to compare this Adjunct Past/Tense marking ratio between the two groups. In addition, a Spearman correlational analysis was performed within both groups to investigate possible relationships between Tense marking and Spatiotemporal, VP-attached, Within-propositional (non-spatiotemporal), Outside-propositional adjuncts, respectively. To test whether a possible effect of Tense marking would be specific to Tense, correlational analysis between Modality and the Adjunct types was run as well.

Furthermore, to test the assumption that the overuse of Quotational embedding which involves predominantly the present tense in BA might have an impact on the use of past Tense morphology, we ran a final correlational analysis between Tense and Embedding (Quotational, Non-quotational as well as the Non-quotational to Quotational ratio) for the clinical group.

### Results of post-hoc analyses

Table 5 shows the mean and standard deviation of Spatiotemporal, non-Spatiotemporal of the Within-propositional adjuncts, temporal adjuncts indicating time reference to the past, as well as the Adjunct Past/Tense marking ratio for the two groups. As seen there, the mean proportion of Spatiotemporal adjuncts was higher than that of the Non-spatiotemporal adjuncts in both groups. The Wilcoxon signed-rank test suggested that PWA have a significantly higher Adjunct Past / Tense marking ratio in relation to the controls.

**Table 5:** Decomposition of within-propositional adjunct.

	Mean ± SD		
	BA	HC	
Spatiotemporal	0.095 ± 0.071	0.214 ± 0.076	
Non-spatiotemporal	0.024 ± 0.032	0.092 ± 0.053	
Temporal Adjunct-Past	0.026 ± 0.025	0.031 ± 0.037	
Adjunct Past / Tense marking	0.177 ± 0.149	0.068 ± 0.087	P = **0.043**

Table 6 displays the results of a post-hoc correlational analysis of Tense in relation to Adjunct types. Spearman tests revealed a moderate positive association between Tense and Spatiotemporal adjuncts for both groups. Tense was additionally correlated with VP-attached adjuncts in the clinical group. In BA, significant correlations were also found between Tense and Non-quotational embedding and Non-quotational/Quotational ratio. No significant correlations were found between Modality and Adjunct types.

**Table 6:** The results of the correlational analysis.

	Tense marking		Modality	
	BA	HC	BA	HC
VP- attached	r = 0.573	r = -0.208	r = 0.055	r = 0.184
	p = **0.013**	p = 0.406	p = 0.828	p = 0.465
non-spatiotemporal	r = 0.245	r = 0.212	r = 0.025	r = 0.447
	p = 0.328	p = 0.399	p = 0.922	p = 0.063
Spatiotemporal	r = 0.491	r = 0.575	r = -0.124	r = 0.162
	p = **0.038**	p = **0.013**	p = 0.624	p = 0.521
Outside-propositional	r = -0.107	r = 0.212	r = 0.360	r = 0.381
	p = 0.672	p = 0.399	p = 0.142	p = 0.119
Quotational embedding	r = 0.020			
	p = 0.938			
Non-quotational embedding	r = 0.693			
	p = **0.001**			
Non-quotational / Quotational Ratio	r = 0.748			
	p < **0.001**			

## Discussion

Our predictions were based on the general hypothesis of a preserved grammatical competence in BA. Lack of such a preservation would have been manifest in underuse of functional morphemes or adjuncts at particular or all heights of the syntactic hierarchy, in anomalous patterns in their distribution relative to each other, anomalous pausing patterns, or error patterns marked by grammatical ‘mistakes’ rather than omissions. Overall, our results contradict this pattern and support our hypothesis.

Specifically, in line with our first specific prediction, there was an absence of group differences in the use of functional morphemes at the hierarchically highest layer of the ATM complex, i.e. Modality, as well as in the lowest layer, Aspect. As Modality is higher than both Tense and Aspect, and a modal presupposes a clausal unit in its scope, this entails a syntactic capacity at the high end of the syntactic tree, suggesting this specific layer of grammatical complexity is no less accessible to this aphasic group. Though Modality is quantitatively less used than the other two variables in the ATM complex, this could in part be due to the morphological differences in the use of modals as independent words vs. use of inflections (as with Tense and Aspect). Like Aspect, moreover, Modality is nothing that is grammatically required. Hence while the PWA could have spared themselves its use, they did not do so more than controls.

Differences between the groups were, however, seen in Tense, in the form of a significant under-use of Past morphology in BA. On the one hand, this result contradicts our prediction for Tense as part of the ATM complex, and it also seems consistent with previous finding using elicitation tasks that people at least with more severe forms of aphasia are not able to access the tense node in the syntactic hierarchy [33], and also with claims of an impairment in reference to the past in PWA [64], and less such impairment in Aspect, which is syntactically lower in the hierarchical phrase structure. On the other hand, this would not explain why Modality, hierarchically higher than Tense, was equally often produced in both groups. Moreover, while markers of temporal displacement were less produced by PWA, the estimated increase in the incidence rate ratio in controls was relatively small (1.74); and previous literature claiming a deficit of reference to the past have not always distinguished between the two notions of use of tense morphology, on the one hand, and of reference to the past, on the other. The latter of these can be realized grammatically in several other ways than with the use of either Past tense or tense morphology (see further below). For example, the experimental design of Bastiaanse [64] does not directly investigate reference to the Past in English, as distinct from the notion of the production of Past tense morphology or present perfect.

A number of factors could contribute to the lower rate of Past marking in BA in our study, which do not relate to Tense per se. One is that in English, Past is often expressed on auxiliaries, and these are often omitted in BA for independent reasons, resulting in characteristic productions like *Dad driving away and pants on the floor; I talking; But shoes not good* (see further below). In the context of telling a story about a past event, the omission of the auxiliary, and hence along with it of Tense, plausibly is one adaptive strategy when seeking to tell such a story at all. An adaptive strategy to circumvent a grammatical constraint may to some extent suggest the awareness of that constraint. Additionally, temporal displacement can be grammatically expressed through temporal adjuncts, as in *That morning…Dad driving away and pants on the floor*. In a population with specific problems of inflectional morphology and agreement, it would make perfect sense to circumnavigate this difficulty by employing this strategy as well. A previous eye-tracking study has already suggested that processing of adverb-verb relations imposes less time cost than that of subject-verb relations [65]. Evidence for this adaptive strategy would be a correlation between use of Tense morphology and temporal adjuncts, as this would indicate that they are related and tap into the same underlying cognitive process. In line with this interpretation, our post-hoc correlational analyses revealed that the quantity of Tense markers was indeed selectively associated with Spatiotemporal adjuncts, but not with Non-spatiotemporal or Outside-propositional adjuncts, for both groups. Additional evidence supporting this perspective was the significantly higher ratio of temporal adjuncts indicating the past to Tense markers (i.e. the Adjunct Past / Tense ratio), in the BA group relative to controls. The examples below illustrate a common pattern for many PWA in the story narrative to use temporal adjuncts to first establish the time frame and then sparing the use of verbal morphology:

1. *October two thousand five*, *coffee*, *five thirty coffee*, *and dog*, *and bathroom*, *and fall down*, *three or four times*, *and Jenn open the door*: *“what wrong*?*”*. *Jenn and Scott just help & = ges*:*support the bedroom*, *and Scott sit down and nine one one*, *and ambulance and Huguley*.
2. *Long time ago*, *years ago*, *a stroke*, *I guess Debbie is with me*. *I remember Debbie is here*. *No*, *she isn’t*. *I guess fall hard*.

In short, under-use of Tense morphology in BA cannot be regarded as evidence *per se* that the Tense layer of the syntactic hierarchy is not activated in BA: it has to be regarded in the context of other variables (e.g. modality, targeting layers above Tense), and of adaptive strategies consistent with the intactness of the T layer (dropping auxialiarties in general, use of temporal adjuncts). As a final piece of evidence in the same direction, many of our subjects with aphasia tended to quote or imitate the characters during the picture description tasks, creating a self-dialogue among these characters (see further on clausal embedding, below). Creating such dialogues using quotational embedding involves predominantly *present* Tense, in a past context: e.g., *Singing in the rain*, *singing in the rain*, *how happy I’m feeling; oh*, *I need a ladder; I’m going to run home and get the umbrella*. Ipso facto, therefore, we would expect less use of Past tense from an increase of quotational embedding. In line with this, correlational post-hoc results showed a significant positive association between Tense and Non-quotational embedding, as well as between Tense and the Non-quotational to Quotational embedding ratio in BA, suggesting that the lesser use of Tense in BA may indeed be in part explained by their lesser use of Non-quotational embedding.

The CP level as indexed through clausal embedding takes us one step higher in the syntactic hierarchy. Our specific prediction here were similar levels of clausal embedding as controls, with a possible compensation effect seen at the level of quotational embedding, in line with previous literature. As Fig 2 shows, embedding alone showed a ‘crossing’ line pattern when comparing Quotational to Non-quotational embedding: PWA significantly *under*used the latter but *over*-used the former. For a typical example, when describing the first two panels of the cartoon picture ‘Refused Umbrella’, which shows a mother trying to give her child an umbrella, most PWA tended to use direct speech like *You have to take the umbrella*!*—No*, *I don’t need it*, *I will be fine*, while healthy controls would use indirect speech or embedded clauses such as *The mother is telling the child to take the umbrella; The son is telling his mother that he doesn’t need it*, *he will be fine*. Quotational embedding is a form of embedding, insofar as an utterance like *Take the Umbrella*! would be uninterpretable (or misinterpreted) if produced without an indication (through intonation, gestures, or other devices) that the utterance is attributed to someone else, and in this sense embedded under a silent or overt marker such as *She said*:… We interpret this pattern as evidence for quotation as a compensatory strategy for canonical clausal embedding when the latter is hard to produce. Harnessing their pragmatic skills to bring their stories to life [45, 66], PWA go for another strategy that grammar avails.

Located even higher in the syntactic hierarchy than the CP-level as targeted through clausal embedding, we investigated interactional language. In line with predictions, the two groups did not differ in the production of Confirmationals. Yet PWA produced a higher proportion of Response markers. This is expected insofar as for those unable to respond to the protocol questions, the examiner would provide prompts, by which the conversation became more interactive, making response markers more likely to be produced. Also, PWA often produced response markers in response to their own speech, e.g. in self-corrections (e.g. *bee*? *no*, *bird; well*, *uh I had a stroke in forty one two three &-uh well*…*no…*). Response markers are a way of keeping the conversation flowing in the face of production problems. It was highly typical of PWA to use them when initializing a topic, first responding to a panel by saying things like *oh*, *the dog is barking; oh*, *he gotta go; oops*, *he is not taking his umbrella* and creating a kind of dialogue facilitating the narrative.

Early studies of aphasic communicative competence have already highlighted the significant role of discourse markers as compensatory strategies to promote social interaction [67, 68]. It is claimed that discourse markers such as *oh*, *well*, *I mean*, *you know*, *okay*, are devices for managing the flow of information or serve as cues to the speaker’s frame of reference and attitude [69]. The successful development of such markers has often been regarded as a crucial aspect of ‘pragmatic’ skills by PWA in their social conversation. As noted, however, current linguistic models capitalize on the fact that the interactional dimension of language forms an inherent aspect of grammatical organization [46, 47, 70, 71]. Response markers and confirmationals can be modelled as a hierarchically organized layer of structure above where propositions are expressed, to which truth values can be assigned [47]. Speakers’ knowledge about constraints on the use of these interactional dimensions of language is, on this view, an integral aspect of their grammatical competence.

Turning to adjunct placement, PWA produced significantly fewer adjuncts of each type in relation to the controls. This is in line with adjuncts as being grammatically dispensable by definition, making them natural candidates for omission in ‘telegraphic’ speech that has to stick to the essentials. Previous eye-tracking experiments on real-time sentence production have also revealed greater processing cost for adjuncts as compared to arguments in PWA, as reflected by longer gaze durations to adjuncts than arguments [72]. Our primary question, however, related to adjuncts in their associated hierarchical positions. Here our study showed that the intercepts of all three types of adjuncts (the predicted values of each type of adjuncts for the clinical group) were highly significant, providing evidence that the amount of each adjunct type PWA produced was significantly different from zero, at all attachment heights. Moreover, when compared with controls, the relative incidence rate ratios for the three types of adjuncts represented an interesting decline from VP-attached (4.02) to Outside-propositional (3.00), and to Within-propositional adjuncts (2.45), implying that differences between groups were smaller in adjuncts with *higher* attachment sites in the syntactic hierarchy. Many PWA used propositional-level adjuncts to express their subjective evaluation about or epistemic attitude towards sentence-level contents, even where they could not produce these contents in sentences that were grammatically fully correct. This pattern is in line with Armstrong and Ulatowska’s [73] finding that PWA were successful in using evaluative language and similar to the controls in the devices used.

Finally, when we zoomed into the relative proportions of each type of adjuncts relative to every other *within* each group looked at separately, the pattern was highly similar across groups, following a downward tendency from Within-propositional adjuncts to Outside-propositional and VP-attached adjuncts (see Fig 2). VP-attached adjuncts are limited to those adverbials modifying lexical verbs in the clause, while Outside-propositional adjuncts mainly involve epistemic and affective adverbs taking scope over the entire proposition. Both of these two types of adjuncts occurred less frequently compared to Within-propositional adjuncts, which often include a variety of phrases adding extra meaning such as time, space, reason, purpose to the clause, with a high proportion taken up by Spatiotemporal adjuncts such as *right here*, *now*, *first*, *then*. The sameness of the pattern in relative proportions of adjuncts at different syntactic heights is further evidence in line with the hypothesis that grammatical knowledge or competence remains intact in PWA.

Altered patterns of speech pauses provide further insights into cognitive mechanisms involved in speech generation. Again, and strikingly, the two groups followed a very similar pattern with regards to the relative proportions of the three pause types relative to each other. In both groups, the proportion of Between g-unit pauses was significantly higher than that of the Between-phrase and the Within-phrase pauses, which corresponds to smaller units of structure. In other words, both groups would invest the most thinking power to assemble larger grammatical units, suggesting the same structural complexity effect, and hence awareness of such complexity, when trying to get it right. Moreover, it was only in Between g-unit pauses that PWA produced more pauses. This finding broadly confirms a finding in a previous study of silent pauses in post-stroke aphasia [55], which documents a qualitatively similar and only quantitively altered pausing pattern in PWA, although this study was focused on pause duration and did not distinguish syntactic positions. The present study further specifies this broad result within a group with BA alone, including filled pauses and based on a fine-grained differentiation of syntactic pause positions. Our basic finding is that the relative proportions of pauses across these positions is neurotypical in BA, and even pause frequencies are confined to utterance-level positions. This comes as a surprise as speech dysfluency is one of the most striking features that define BA.

Finally, as anticipated, omissions were the most prominent among the error types we distinguished (see also [74]). Importantly, while some types of word order violations could be suggestive of a distortion of the syntactic hierarchy, omissions of the type found here are not. Prominent among the early interpretations of omissions is a principle of economy: the effort involved in the production of speech causes the patient to reduce his output to the barest information-carrying words of the message [74]. Omissions, moreover, are a strategy to avoid errors of a kind that the speaker knows would arise if some morphological item was attempted to be put in place. In turn, morphological errors, whose proportion accounted for a relatively small proportion, are suggestive of a problem with morpho-syntactic specificities of a particular language (e.g. determiners in English), more than the loss of grammatical knowledge, which as such would apply to any language. The overall error pattern is consistent with Kolk & Heeschen’s [75] proposal that the characteristic ‘agrammatic’ speech seen in people with BA represents an adaptation symptom, resulting from a coping strategy rather than the deficit in knowledge itself.

Overall, like the access hypothesis of Hula & McNeil [3], our study throws doubt on BA as a model of a loss of grammatical competence and hence linguistic impairment at this level. At least at the level of severity targeted here, PWA do, in the words of Friedmann [33], ‘climb up the syntactic tree’–and the entire one. Hierarchy is not the problem. Yet this does not entail, or need to entail, that their productions will be formal-grammatically correct at a morpho-syntactic level. Rather, it reinforces the need for appropriate models of the language deficits seen in BA, which would have to identify mechanisms explaining these deficits while taking into account that the syntactic hierarchy as such may be intact, and adaptive strategies may be at play. Mechanisms of agreement [65], intervention effects [76] and feature impoverishments [77] should all be considered in this respect. Such models should be tested both from the viewpoint of experimental sentence completion tasks [33] and of patterns detectable in spontaneous speech as studied here.

These conclusions have further foundational implications as well as practical ones at a clinical level. The former arises in the context of a traditional claim that aphasia is a model of ‘thought without language’ [25], leaving thought broadly intact while language is impaired. Both the present results and the broader empirical patterns supporting versions of the access hypothesis suggest that this modularist claim could not be based on a failure of grammatical competence in BA. On the contrary, they invite the idea that insofar as thought is intact in aphasia, this could be in line with the intactness of grammar at a competence level. This might be further supportable by specific linguistic views of the syntactic hierarchy as effectively mirroring hierarchical layers of semantic complexity, possibly with no clear distinction between these two [66, 78]. These would naturally suggest that the putative intactness of thought in PWA is actually a reflection of the intactness of grammar at this syntactic-semantic level. Clinically, our results bear on defining treatment targets in aphasia, which should seek to harness and bring out the grammatical competence retained in BA. More broadly, they also bear on the public and stigmatizing image of BA as ‘agrammatism’.

### Limitations

Our results are based on a limited window into the spectrum of BA given our recruitment criteria, which excluded PWA and less than three-word utterances from this study, without which our annotation scheme would not have been applicable. Our results therefore do not generalize to BA at large. It is possible that an inclusion of severe cases could have profound implications for a deeper understanding of language-cognition links, and specifically reveal that where grammatical competence *is* indeed lost, distinctive cognitive effects would be observable alongside.

## Conclusion

This is the first study to examine hierarchical grammatical complexity in spontaneous connected aphasic speech. The overall pattern of results questions a loss of grammatical competence as operationalized through variables across different domains of the syntactic hierarchy, at least in those people with BA that produce minimal sentences at all. This in turn questions the idea of BA and/or agrammatism as a primary disease model of grammatical competence. Future studies could gain further insights from typologically different languages and the inclusion of PWA at the severe end of the scale, specifically to explore relations between language and cognition when language is at even more minimal levels.

## Supporting information

S1 File Main linguistic variables (Table 1) and Error subtypes (Table 2). (DOCX)

S1 Dataset (XLSX)
