# Supplementary material for: Grammar in ‘agrammatical’ aphasia: What’s intact?
Source: PLoS One. 2022 Dec 6;17(12):e0278676. doi: 10.1371/journal.pone.0278676 (PMC9725141; doi:10.1371/journal.pone.0278676)
Supplement: S1 File — (DOCX) [file pone.0278676.s001.docx]

| **Supplementary Table 1.** Main linguistic variables | | | | | | | |
| --- | --- | --- | --- | --- | --- | --- | --- |
| Variable | | Definition | | | Example (from transcripts) | | |
| Sentential g-unit | | Sentence-type grammatical meaningful unit. | | | Fully grammatical g-unit：  *It looks like this little boy is kicking the ball.*  G-units including formal-grammatical errors:  *My father has blind; I have sell my boat; Now let’s me finish; this side don't working.* | | |
| Non-sentential g-unit | | Isolated words or phrases which make a meaningful contribution to the discourse. | | | *wonderful; married; finally; lamp; fireman; tree; raining oh boy splashing;*  *all of them; three times a day; five weeks; in the afternoon.* | | |
| Aspect | | Grammatical marking of how an action or event denoted by a verb extends over time relative to the point of speech (e.g. progressive, inchoative, habitual, perfective). | | | *My boyfriend is watching TV.*  *and they had closed the street to traffic.*  *I used to speak a lot of Spanish.* | | |
| Tense | | Past tense marking including Simple Past, Past Continuous, Past Perfect, Past Perfect Continuous. | | | *and he went out.*  *The boy was kicking his ball.* | | |
| Modality | | Grammatical marking of possibilities, necessities, abilities, permissions, norms, etc*,* usually through auxiliaries. | | | *He can't climb into the branches or something.*  *I'm able to say it but hard.*  *I need a cane.* | | |
| Quotational embedding | | Someone’s utterance is repeated as indicated through embedding under speech-act verbs (e.g. *he said:…*), intonation, or context. | | | *and he looked around and he said "oh I need a ladder".*  *“you're gonna get it”.^[[1]](#footnote-1)^*  *“I don’t need it. I will be fine”.* | | |
| Non-quotational embedding | | Embedded clauses subordinated under a matrix verb. | | | *I don't think I have anything.*  *it looks like this little boy is kicking the ball.* | | |
| Response marker | | Response markers in response to polar questions, or in response to all kinds of speech act, e.g. wh-questions, exclamatives, assertions. | | | ***yeah, well****, I saw my grandson.* (In response to the investigator)  *‘****Oh****, I see.’* (In response to himself) | | |
| Confirmational | | Confirmationals such as *you know, I mean, isn’t it.* | | | *ladder,* ***yeah****.*  *so,* ***I mean****, it was nice.*  *and* ***you know*** *I try to talk whenever I can to help me.* | | |
| VP-attached adjunct | | Adverbials attached to VPs that modify the event/action (e.g. slowly, eagerly). | | | *I guess fall* ***hard****.*  *I don't* ***officially*** *remember all that stuff.* | | |
| Within-propositional adjunct | | Adjuncts adjoining to larger units such as clauses with scope within a propositional (truth-evaluable) unit (e.g. spatio-temporal adjuncts like *in the afternoon, five years ago*, *on the floor*, or purpose clauses, e.g. *to find the cat*). | | | *three times a day; many years ago;*  *he came up here* ***to find the cat****.*  *and I was unconscious* ***on the floor****.*  *and so I was in the hospital* ***for a week****.* | | |
| Outside-propositional adjunct | | Adjuncts adjoining to larger units such as clauses with scope outside a propositional unit. For example, adjuncts expressing a speaker’s evaluation of a proposition, e.g. *Sadly, Surprisingly, Stupidly*, or his epistemic or evaluative relations to a proposition, e.g. *Actually, Really interesting!, Amazing!* | | | *and the father* ***clearly*** *tried to go up the tree and get it.*  *it's* ***probably*** *the daddy.* | | |
| Between g-unit pause | | Pauses that occur in a boundary position of the g-unit (Specifically, at the beginning of the g-unit). | | | ***&-um*** *I was working.*  ***&-um*** *it was on a Sunday.* | | |
| Between-phrase pause | | Pauses that occur within the g-unit, and occur between the subject and the predicate or between the matrix clause and the subordinate clause. | | | *and then they* ***&-um &-uh*** *discharged me.*  *and when I laid down* ***&-um*** *the right side of my body was paralyzed.*  *and &-um I went back to speech* ***&-um*** *after I was discharged.* | | |
| Within-phrase pause | | Pauses that occur within a g-unit, and specifically within single phrases. (e.g. between the determiner and the noun in NPs; between the auxiliary and the verb; between the preposition and the NP/VP; between the copula and the adjective/noun; between the main verb and the object; or between the adverb and the verb). | | | *so, he went into* ***&-um*** *his* ***&-um*** *bedroom and laid down .*  *and I was* ***&-um*** *twenty.’*  *and he was* ***&-um*** *covering his head.* | | |
| **Supplementary Table 2.** Error subtypes | | | | |  |  |  |
| Error Types | | Definition | Example (from transcripts) | | |  |  |
| Omission | | Omissions of free morphemes such as determiners; auxiliaries; prepositions; verbs, nouns, or bound morphemes such as agreement and tense morphemes. | *dog is barking.*  *my father has &-um blind.*  *my mother said I told you to the umbrella.*  *I am hospital.*  *ball cracking the window.*  *the basketball* *get in the way.*  *and the window it open.* | | |  |  |
| Morphological error | | Wrong morphology, e.g., errors in verb inflection; auxiliaries. | *Now lets me finish.*  *but I doesn't speak clearly.*  *man is shattered a ball.* | | |  |  |

| Word order violation | Violation of the typical Subject-Verb-Object word order, or violation of word order within phrases (e.g. the reverse of determiner and noun in NP). | *I have really problems with &=shows:arm.*  *man a fall down.* |
| --- | --- | --- |

1. This utterance was produced in the picture description task ‘Refused Umbrella’. The participant was mimicking the mother who is asking his son to take the umbrella with him because it’s raining outside. [↑](#footnote-ref-1)
